# Methods for Prioritizing Causal Genes in Molecular Studies of Human Disease: The State of the Art

**DOI:** 10.1002/gepi.70037

**Published:** 2026-03-02

**Authors:** Karina Patasova, Bahar Sedaghati‐Khayat, Rachel Knevel, Heather J. Cordell, Arthur G. Pratt

**Affiliations:** ^1^ Translational & Clinical Research Institute, Faculty of Medical Sciences Newcastle University Newcastle upon Tyne Tyne and Wear UK; ^2^ Department of Rheumatology Leiden University Medical Center Leiden South Holland Netherlands; ^3^ The Delft Bioinformatics Lab, Pattern Recognition and Bioinformatics Delft University of Technology Delft South Holland Netherlands; ^4^ Population Health Sciences Institute, Faculty of Medical Sciences, International Centre for Life Newcastle University Newcastle upon Tyne Tyne and Wear UK; ^5^ Musculoskeletal Unit Newcastle upon Tyne NHS Foundation Trust Newcastle upon Tyne Tyne and Wear UK

**Keywords:** causal inference, causal network, colocalization, Mendelian randomization

## Abstract

In the last decade, genome‐wide association studies (GWAS) have identified tens of thousands of common variants associated with a wide array of complex traits and diseases. Integration of GWAS with molecular data has informed the development of statistical tools for causal gene discovery. In this paper, we give an overview of commonly used causal inference methods and discuss the strengths and limitations of colocalization, Mendelian randomization (MR) and network‐based approaches. Colocalization is often used to assess whether the genetic association signals for two traits arise from the same causal variant, thereby strengthening inferred causal associations. MR was developed to tackle issues of confounding and reverse causality, providing a rigorous approach to causal inference and demonstrating improved false discovery rates. Unlike MR, network‐based analyses employ a discovery approach and model complex relationships between multiple variables. All causal inference methods are, to varying degrees, susceptible to spurious associations due to genetic confounding, pleiotropy and linkage disequilibrium. Here, we discuss the latest developments in the field of causal gene inference and limitations of these methods. We give an overview of interplay between different approaches as well as practical applications with reference to published examples in context of heart disease.

## 1 Introduction

Genome‐wide association studies (GWAS) have identified tens of thousands of genetic associations with complex traits and diseases, revealing that these conditions are often associated with multiple independent common polymorphisms (Uffelmann et al. 2021). This approach has provided insights into the allelic architecture of complex traits and suggests potential genetic heterogeneity among affected individuals. However, up to 90% of GWAS‐identified variants lie in non‐coding regions with some of these associations affecting gene expression (Uffelmann et al. 2021). However, by and large, the functional impact of intergenic markers remains unclear. Moreover, GWAS signals typically include lead variants that tag many correlated SNPs in a specific genomic region and are not necessarily causal markers. Disease‐associated genetic loci often harbor multiple mechanistically plausible genes, many of which could be causal based on proximity alone; this makes it difficult to infer causal candidates from based on GWAS findings (Uffelmann et al. 2021).

In the context of post‐GWAS analyses, the term “causal gene” denotes the gene at an associated locus through which, by its function, sequence variation directly impacts trait etiology (Costanzo et al. 2025). Although “causal” or “causative gene” is commonly used in casual inference literature, it has been argued that “effector gene” may be a more appropriate term because (i) it does not suggest certainty or deterministic causality (Costanzo et al. 2025), which can rarely be proved using the high‐throughput technologies typically deployed by researchers, and (ii) because it is more precisely the *sequence variations* themselves that are *causal*, leading to observable changes in gene function with their etiological consequences (Costanzo et al. 2025). Whilst acknowledging this point, we use the term “casual gene” in this review for consistency.

To address inherent complexities of causal inference for candidate gene prioritization, an array of methods has been developed. These methods systematically integrate GWAS and multi‐omics data, including gene expression, metabolomics, and proteomics, thereby expediting biomarker profiling and discovery (Cui et al. 2022). Omics refers to a comprehensive study of biological molecules, which include genes, transcripts, proteins, methylation and metabolites (Song et al. 2020). Quantitative trait loci (QTL) are regulatory regions that contain genetic variation influencing the levels of various omics molecules, frequently through genetic and gene‐environment interactions (Powder 2020). QTL‐based causal inference methods are widely used and can be broadly classified as colocalization analyses, instrumental variable (IV) approaches, and network‐based causal frameworks (Chen et al. 2024; Zuber et al. 2022; Yin et al. 2024).

Establishing causality in complex biological systems requires a careful consideration of potential confounding factors. Colocalization analysis serves as a crucial preliminary step in a larger causal inference pipeline that establishes whether GWAS and QTL signals in a given genomic region share a single causal variant or result from multiple (separate) causal variants (Giambartolomei et al. 2014; Wu et al. 2019). By statistically determining the likelihood of shared causal markers, colocalization can facilitate selection of instrumental variables (IVs) for subsequent causal inference methods but also can be used to refine causal associations with effector genes (Zuber et al. 2022).

IV analysis, with Mendelian randomization (MR) being the most prominent example, utilizes genetic variants as uncounfounded instruments (IVs) to establish causal relationships between an exposure (such as gene expression) and an outcome (such as disease risk) (Chen et al. 2024). Unlike observational studies that are prone to residual confounding and reverse causation, MR exploits Mendel's law of random assortment of alleles at conception, thereby creating conditions similar to a randomized control trial (Chen et al. 2024). This principle allows MR to identify candidate genes by explicitly testing whether predicted changes in biomarker levels have a causal influence on a particular trait (Zhu et al. 2016).

It is crucial to differentiate MR from related but separate approaches such as transcriptome‐wide association studies (TWAS) (de Leeuw et al. 2023). While both methods leverage GWAS and multi‐omics data to prioritize candidate genes, TWAS surveys genes whose genetically predicted expression levels are associated with the trait (de Leeuw et al. 2023), using the same conceptual framework as MR but without formally acknowledging (or attempting to mitigate) any violations of IV assumptions. Generally, TWAS findings should not be interpreted as evidence of causality due to inherent limitations of this method, namely, genetic correlation, linkage disequilibrium and horizontal pleiotropy (de Leeuw et al. 2023). The role of TWAS in the broader causal inference framework, including MR, will be discussed later as part of the MR section of this review.

Beyond hypothesis‐driven approaches like MR, network‐based causal inference analyses offer an alternative discovery‐oriented framework for understanding complex biological systems (Yazdani et al. 2022). In particular, network‐based causal inference methods are able to reconstruct key regulatory hubs and causal pathways by inferring causal and mediatory relationships from high‐dimensional multi‐omics data (Yazdani et al. 2022). MR principles are embedded in the network construction with nodes representing different variables (including genetic variants) and edges denoting directional relationships (Yazdani et al. 2022).

In this review, we set out to give a comprehensive synthesis of state‐of‐the‐art causal inference methods and their application in gene prioritization. We discuss fundamental principles of MR, colocalization and network‐based causal inference approaches, outlining their strengths, limitations, and complementary roles. The objective of this paper is to guide readers in strategically combining these methodologies to enhance the accuracy and robustness of statistical causal inference, thereby fostering a more comprehensive perspective on gene prioritization in post‐GWAS analyses. Concluding each section, we present examples of methodologically sound applications of causal inference, contrasting them with exemplars of key methodological challenges encountered in causal reasoning research.

## 2 Colocalization

### 2.1 Historical Context and General Use Cases

**Figure 1:**
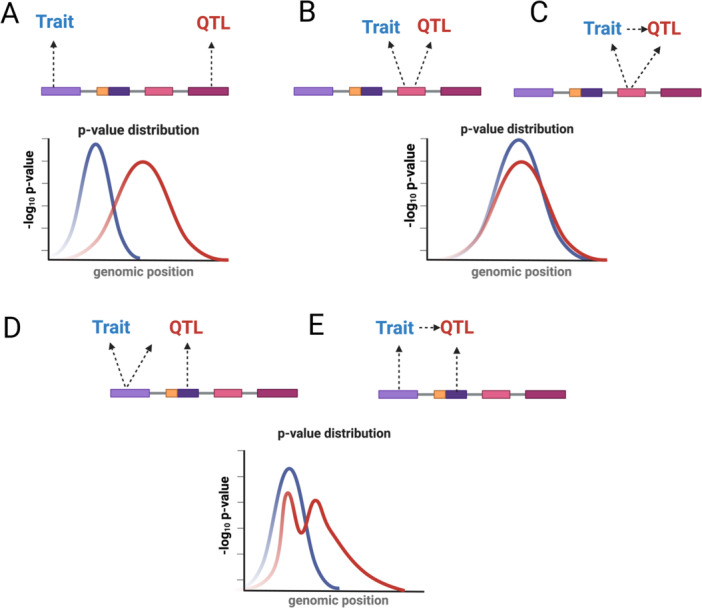
Five different colocalization scenarios. Panel A shows a scenario where a trait of interest and a QTL involve two distinct causal variants in a linkage disequilibrium. Panels B and C display colocalization. In panel B, a trait and QTL are independent but share a single common causal variant. Panel C shows a situation where a trait and QTL also share a single common causal variant, but the trait influences the QTL. Panel D and E display the situation where the trait and QTL have a shared causal variant in conjunction with distinct causal variants. Traditional colocalization methods cannot differentiate between situations where the trait and QTL are unrelated (B and D) and those where either the trait or QTL influence each other. Figure adapted from Zuber V et al. “Combining evidence from Mendelian randomization and colocalization: Review and comparison of approaches” (Zuber et al. 2022). The figure was produced in Biorender.

Genetic markers that are located near each other on the genome are usually inherited together and are strongly correlated (i.e. are in linkage disequilibrium (LD)) (Zuber et al. 2022). LD presents a challenge when analyzing a large number of genetic associations, as two traits may be associated with distinct variants that are correlated with each other (Zuber et al. 2022). Colocalization methods were introduced to help address the lack of mechanistic clarity from GWAS and determine whether different traits (such as phenotypic outcome and gene expression) share causal markers (Zhang et al. 2022) (Table 1). Colocalization is often used in conjunction with MR analyses and can help evaluate the validity of MR assumptions in a given genetic locus. Colocalization assesses the overlap between causal variants for two or more traits by considering alternative scenarios at the locus: two traits with distinct causal variants in LD with each other vs a single shared association signal (colocalization) (Figure 1) (Hukku et al. 2021).

**Table 1:** Colocalization methods.

Method	Description	Strengths	Limitations
Colocalization Tests of Two Genetic Traits (coloc) (Giambartolomei et al. 2014); Hypothesis Prioritization for multi‐trait Colocalization (HyPrColoc) (Foley et al. 2021)	Leverages the Bayesian framework to estimate posterior probability of trait and eQTL sharing a causal SNP within the same locus. Only infers colocalization when there is compelling evidence supporting it.	Computationally efficient and can be applied across multiple loci; uses a Bayesian framework that allows prior specification, generating interpretable probabilities rather than binary decisions; can be extended to multi‐trait colocalization, using HyPrColoc.	Assumes one causal variant per trait per region, does not directly model LD; requires overlapping SNPs between datasets; sensitive to prior specifications.
Sum of Single Effects (SuSiE); coloc. susie (Wallace 2021; Zou et al. 2022)	Conducts fine‐mapping by breaking down genetic associations into sum of single effects processed in sparse model fitted for each trait separately. Separates statistical evidence of association for each variant while conditioning on causal signal at the locus.	Improves resolution for loci with allelic heterogeneity; LD structure is incorporated in the model; aids prioritization by quantifying probability of SNP being causal.	Assumes additive and linear effects; misestimation of number variants in credible sets or inaccurate LD matrix can lead to misleading results.
Expression Quantitative Trait Loci and GWAS Colocalization Analysis via Integrated Association Rates eCAVIAR (Hormozdiari et al. 2016)	Specifically designed to detect colocalization between GWAS and eQTL data by calculating colocalization posterior probabilities.	Models multiple causal variants at the locus by enumerating different causal variant configurations and computing joint probabilities; can detect loci where causal variants are different in GWAS and eQTL data; provides a confidence interval for colocalization of GWAS variants; functional annotations can be used to improve prior specification and fine‐mapping; incorporates LD matrix to account for the correlated structure between SNPs.	Computationally intensive; requires accurate ancestry‐matched LD reference panel.
Colocalization and Fine‐mapping in the presence of Allelic Heterogeneity (CAFEH) (Arvanitis et al. 2022a)	Implements a hierarchical Bayesian model to perform fine‐mapping and colocalization across multiple traits.	Detects shared and distinct causal variants across multiple traits, tissues and cell types, thereby increasing statistical power; models allelic heterogeneity and is able to identify multiple causal variants at the locus.	Computationally intensive due to Bayesian inference procedure; requires accurate ancestry‐matched LD reference panel; assumes equal prior probability for all variants at the locus; unable to detect the presence of multiple causal association signals at the locus that has variants in high LD; doesn't allow missing values in effect size matrix.
Shared sparse Projection for colocalization analysis (SharePro)	Implements effect group‐level approach for colocalization	Handles allelic heterogeneity and LD structure by grouping correlated variants and assessing colocalization at effect group level.	High prior colocalization probabilities increase rates of false positives; requires ancestry‐matched LD reference panel.
Colocalization Quantitative Trait Loci Analysis (ColocQuiaL) (Chen et al. 2022)	Platform that simplifies and streamlines colocalization analysis at scale.	Automates colocalization analyses; accepts a variety of summary statistics formats.	Assumes a single causal variant at the locus; doesn't perform multi‐trait colocalization.
Easy Quantitative Trait Loci (ezQTL) (Zhang et al. 2022)	Platform that simplifies and streamlines colocalization analysis at scale.	Hosts GWAS and QTL public datasets; performs data quality control, LD visualization and paired colocalization analyses; allows multi‐locus query.	Uses a multi‐trait statistical colocalization analyses approach that assumes a single shared causal variant at the locus.

### 2.2 Core Approaches

Colocalization approaches can be subdivided into two major families: proportional and enumeration methods. Proportional colocalization tests the hypothesis that a sole causal variant accounts for genetic associations with distinct traits: proportionality of GWAS and eQTL regression coefficients supports this hypothesis, but can also indicate that the two traits belong to the same causal pathway (Zuber et al. 2022). Enumeration colocalization leverages a Bayesian framework to estimate the posterior probability of a trait and eQTL sharing a causal SNP. One advantage of the enumeration approach is that it only infers colocalization when there is strong evidence supporting it. In the absence of strong evidence, the posterior probabilities will be drawn towards the prior probabilities in order to minimize false positive associations (Zuber et al. 2022).

### 2.3 Relationships With Other Causal Inference Methods

**Figure 2:**
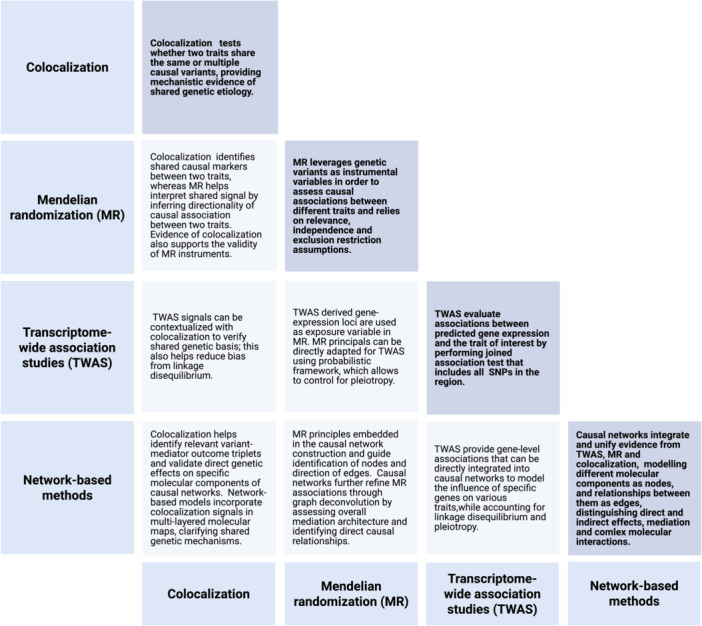
Interplay between different causal inference methods. Figure was produced in Biorender.

Within the broader landscape of causal inference, colocalization holds a distinct yet complementary position alongside other established analytical approaches. When integrated with other methods, colocalization enhances specificity and mechanistic understanding of genetic associations with complex traits. Colocalization is commonly used to contextualize TWAS and MR signals and verify shared genetic basis between gene expression and specific trait (Zuber et al. 2022). Colocalization can also help validate specific components of causal networks (Aygün et al. 2023). The precise interplay between colocalization and other causal inference approaches will be discussed in later sections (Figure 2).

### 2.4 Recent Advancements in Colocalization

Some more recent developments in the area of colocalization include changes to the single causal variant assumption of the original Colocalization Tests of Two Genetic Traits (coloc) method (Giambartolomei et al. 2014), such that the causality of multiple variants within the same locus can be tested simultaneously (Wallace 2021) (Table 1). For example, sum of single effects (SuSiE) carries out fine‐mapping by breaking down genetic associations into the SuSiE that are later processed in a sparse model fitted for each trait separately (Wang et al. 2020; Zou et al. 2022). SuSiE separates the statistical evidence of association for each variant while conditioning on causal signal at the locus (Zou et al. 2022). By contrast, expression quantitative trait loci (eQTL) and GWAS Causal Variants Identification in Associated Regions (eCAVIAR) calculates posterior probabilities, using a model that allows an arbitrary number of causal markers in the locus and considers all possible causal SNP combinations (Hormozdiari et al. 2016). The Colocalization and Fine‐mapping in the presence of Allelic Heterogeneity (CAFEH) method implements a hierarchical Bayesian model to perform fine‐mapping and colocalization across multiple traits (Arvanitis et al. 2022a). Shared sparse Projection for colocalization analysis (SharePro) method combines LD modelling and colocalization by aggregating correlated markers into effect groups (Zhang et al. 2024) (Table 1).

Allelic heterogeneity (Wu et al. 2019), where multiple genetic markers within the same locus are associated with the same trait, presents a significant challenge to colocalization analysis. In the presence of allelic homogeneity, colocalization approaches that assume a single causal variant may erroneously infer colocalization. The presence of multiple causal variants at the locus can potentially reduce the precision of colocalization analyses by diluting the causal association signal and making it harder to pinpoint the exact genetic cause. Similarly, misspecification of prior enrichment levels has also been shown to contribute to spurious colocalization findings (Hukku et al. 2021). Therefore, it has been recommended that prior enrichment levels should be estimated from observed data, and fine‐mapping and colocalization analyses should be carried out separately (Hukku et al. 2021). Careful consideration of analytical factors, such as prior specification and model assumptions, as well as the use of complementary methods, including TWAS and MR, can improve the reliability of colocalization results (Al‐Barghouthi et al. 2022; Rasooly et al. 2023). Platforms such as Colocalization Quantitative Trait Loci Analysis (ColocQuiaL) and Easy Quantitative Trait Loci (ezQTL), further simplify and streamline colocalization analysis at scale **(**Zhang et al. 2022; Chen et al. 2022) (Table 1).

### 2.5 Practical Scenarios

#### 2.5.1 Scenario 1—Colocalization Performing Well

The study by Franceschini et al. on carotid intima‐media thickness (CIMT) and carotid plague integrated GWAS with eQTLs across multiple human tissues (Franceschini et al. 2018). Gene regulation has been shown to be tissue‐specific (Arvanitis et al. 2022b) and impact the accuracy, interpretability and biological relevance of colocalization. Given that CIMT and plaque are pathologies of the arterial wall, strategic selection of arterial tissue as a target eQTL in colocalization analyses allowed the authors to directly link genetic associations to changes in expression in the primary affected tissue (Franceschini et al. 2018). Colocalization signals observed in disease‐relevant tissue supported biological interpretability and strengthened the evidence that candidate genes influenced atherosclerosis progression (Franceschini et al. 2018). Additional conditional analyses were employed to identify independently associated variants; this step ensured that colocalization signals weren't erroneously attributed to distinct genetic markers in LD (Franceschini et al. 2018). To better outline potentially causal associations, colocalization signals were further prioritized using functional annotation and linking CIMT and carotid plague to hard clinical endpoints such as CAD and various types of strokes (Franceschini et al. 2018). By establishing genetic correlations between CIMT and carotid plague and other cardiovascular outcomes, the study strengthened the argument that genes identified though colocalization were relevant for overall risk of severe cardiovascular events (Franceschini et al. 2018).

#### 2.5.2 Scenario 2—Colocalization Producing Potentially Misleading Results

Shandrina and colleagues performed a study that sought to prioritize genes associated with coronary artery disease (CAD) (Shadrina et al. 2020). This investigation highlighted some of the limitations of colocalization analyses (Shadrina et al. 2020). The authors acknowledged that low statistical power and strict significance thresholds, especially in case of weak or marginally significant colocalization signals, might have contributed to false negative associations (Shadrina et al. 2020). Another limiting factor was that per SNP samples were not available and eQTL effect sizes were estimated from Z‐scores, which might have introduced additional variation and affected the accuracy of estimated effect sizes (Shadrina et al. 2020). Incomplete or suboptimal data can hinder the ability of colocalization to detect true causal signals, as the majority of current colocalization methods necessitate full summary statistics for both traits (King et al. 2021). In addition to incomplete summary statistics, another challenge of colocalization pertained to multiple causal associations and complex LD structure. As acknowledged by study authors, in scenarios where multiple causal variants were in a strong LD at particular locus, it was difficult for traditional colocalization methods to discern which variant was a source of colocalization signal (Shadrina et al. 2020).

## 3 Mendelian Randomization

### 3.1 Historical Context and General Use Cases

In biomedical research, genetic polymorphisms are often employed as IVs based on the principles of Mendelian genetics which assert that parental matings and the transfer of alleles from parent to the offspring occur randomly. Thus, individuals in the population can be divided into subgroups based on their genetic risk in a way that is comparable to randomization in randomized control trials (RCTs) (Hingorani and Humphries 2005). The MR approach was first proposed by Gray and Wheatley (Gray and Wheatley 1991), who developed this method in order to tackle some of the methodological issues associated with observational studies such as residual confounding and reverse causation (Chen et al. 2024; Sanderson et al. 2022; Khasawneh et al. 2022). Initially, MR was limited to individual‐level data which included genotypes, exposures and outcomes measured within the same dataset, also known as “one‐sample MR” (Zuber et al. 2022). Subsequent advancements included the incorporation of summary statistics (beta coefficients and their standard errors) from published GWAS, signifying genetic associations between exposure and outcome (Zuber et al. 2022). Introduction of this “two‐sample MR” approach enabled derivation of IVs associated with exposure and outcome from different datasets (Zuber et al. 2022), leading to widespread use of MR with thousands of MR studies currently published (de Leeuw et al. 2022a).

### 3.2 Core Approach

**Figure 3:**
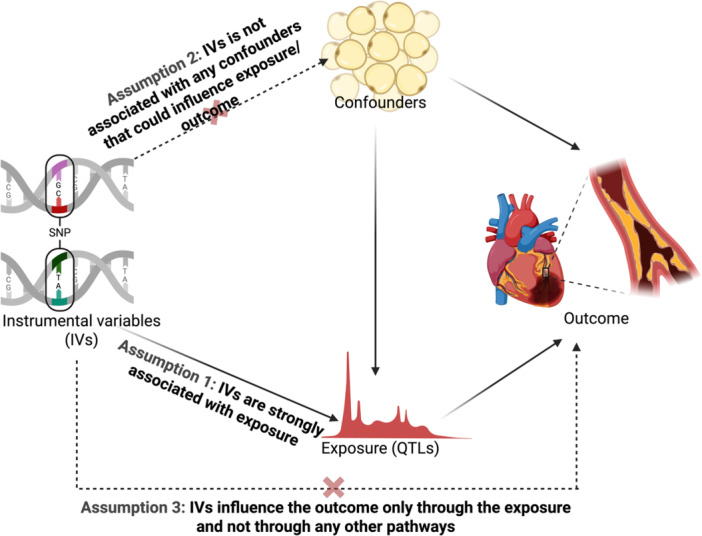
Overview of principles and core assumptions of Mendelian Randomization. Figure was produced in Biorender.

The validity of MR rests on three core assumptions (de Leeuw et al. 2022a) (Figure 3):

a. Relevance assumption: the IV is robustly associated with exposure either directly or through LD with a causal variant.
b. Independence assumption: There are no measured or unmeasured confounders influencing the IV or the outcome.
c. Exclusion restriction: the IV is not directly associated with an outcome but influences it only indirectly through the exposure. This assumption instrument strength is independent of the direct effect.

Additional MR assumptions include:

a. The Instrument Strength Independent of Direct Effect (InSIDE) assumption: IV‐exposure association is not correlated with any direct effects that IV has over the outcome (de Leeuw et al. 2022b).
b. Homogeneity assumption: The effects of IVs on the exposure should be consistent across different subgroups (Small et al. 2017).
c. No Measurement Error (NOME) assumption: There should be no measurement error in IVs (Bowden et al. 2016a).

MR can be implemented in several ways depending on available data and the studied exposure. In one‐sample MR a two‐stage least squares method can be used to assess the causal effect of a particular exposure (e.g. eQTL) on an outcome of interest (e.g. myocardial infarction). The classical MR Wald ratio test provides a causal estimate for a single IV by calculating the ratio of outcome and exposure beta coefficients (Burgess et al. 2017). Inverse‐variance weighted variance (IVW) test aggregates ratio estimates of several IVs in a fixed effects meta‐analysis, weighting each ratio by the inverse of its variance (Burgess et al. 2013; Bowden et al. 2019). In the presence of horizontal pleiotropy, which describes phenomena where a genetic variant (IV) is independently associated with both exposure and the outcome of interest, IVW (Burgess et al. 2013) estimates of this nature may be biased; hence, SNPs contributing to heterogeneity are usually excluded. Although alternative tests, such as weighted median (Bowden et al. 2016b), MR‐Egger (Bowden et al. 2015), and model‐based MR, are comparatively less well‐powered, they can evaluate the validity of IVs and produce more robust estimates of causal effect (Sanderson et al. 2022). See Table 2 for a listing of commonly used MR approaches that are described in this review.

**Table 2:** Mendelian randomization methods.

Method	Description	Strengths	Limitations
Wald Ratio test (Burgess et al. 2017)	Provides a causal estimate for a single IV by dividing the beta coefficient of SNP‐outcome association by the beta coefficient of SNP‐exposure association.	Easy to implement and interpret.	Sensitive to pleiotropy and weak instrument bias.
Inverse variance weighted (IVW) (Burgess et al. 2013)	Combines Wald ratio estimates in a fixed effects meta‐analysis, weighting each ratio by the inverse of its variance. Assumes no horizontal pleiotropy and is sensitive to weak instrument bias.	Increased statistical power.	Sensitive to SNP heterogeneity pleiotropy and instruments invalidity.
Weighted Median (Bowden et al. 2016b)	Estimates the causal effect as the median of the weighted distribution of ratio estimates. Requires that at least 50% of the weight comes from valid instruments.	Less sensitive to outliers with pleiotropic effects than IVW.	Requires a large number of IVs and assumes that 50% are valid.
MR‐Egger (Bowden et al. 2015)	Aggregates Wald‐ratio estimates into meta‐regression, while adjusting for directional pleiotropy. Intercept term is used to test for pleiotropy under InSIDE assumption.	Detects and handles pleiotropy by including an intercept term in the regression model	Less statistically powered than IVW test, sensitive to measurement error.
Summary data‐based Mendelian randomization (SMR) (Zhu et al. 2016)	Aggregates summary data from independent GWAS and eQTL studies to ascertain genes whose expression levels show associations with a trait due to pleiotropy.	Handles large molecular data and identifies genes whose expression levels influence trait.	Can't distinguish between vertical and horizontal pleiotropy and make conclusions about causal associations; is limited to gene expression and doesn't include other mechanisms such as post‐translational modifications; relies on the quality and resolution of GWAS.
Mendelian randomization with linkage disequilibrium and pleiotropy (MR‐link) (van der Graaf et al. 2020)	Employs eQTL summary statistics and individual level data for exposure and outcome, correcting for LD and unobserved pleiotropy without removing pleiotropic IVs.	Explicitly models LD and unobserved pleiotropy without needing to remove pleiotropic variants; derives robust estimates even with small number of IVs.	Requires individual level data; computationally intensive due matrix and LD modelling.
Effective‐Median‐based Mendelian randomization (EMIC) (Jiang et al. 2022)	Identifies false‐positives due to LD and correlation between IVs, using multiple cis‐eQTLs for pleiotropy fine‐mapping. Employs eigenvalue decomposition matrix estimated from ancestry matched reference panel.	Explicitly models LD between IVs and mitigates pleiotropy by using a median‐based estimator without needing to exclude pleiotropic variants.	Requires ancestry‐matched reference panel; computationally intensive due to eigenvalue decomposition of LD matrix; relies on the quality and resolution of GWAS.
Mendelian randomization with correlated horizontal pleiotropy (MR‐Corr^2^) ^(^Cheng et al. 2022a ^)^	Designed to tackle correlated horizontal pleiotropy where genetic variants influence both exposure and outcome variables through shared biologic pathways	Handles correlated horizontal pleiotropy by incorporating a correlation parameter in MR model; allows to use correlated IVs rather than requiring LD pruning by building LD matrix.	Performs under assumption that pleiotropic effects are either entirely correlated or uncorrelated; assumes that only a small proportion of IVs have pleiotropic effects; doesn't account for sample overlap. between exposure and outcome datasets; computationally intensive due to requiring correlation matrices; relies on the quality and resolution of GWAS.
Causal Analysis Using Summary Effect estimates (CAUSE) (Morrison et al. 2020)	Explicitly models both correlated and uncorrelated pleiotropic effects, mitigating reverse causation and weak instrument bias.	Model unobserved pleiotropy and doesn't require prior information about shared heritable factors	LD pruning may exclude informative variants in regions with high LD; doesn't allow to include known shared factors (e.g., measured confounders or mediators) in the model; relies on the quality and resolution of GWAS.
Mendelian randomization with correlated horizontal pleiotropy unraveling shared etiology and confounding (MR‐CUE) (Cheng et al. 2022b)	Considers both shared and IV‐specific correlated horizontal pleiotropy effects. Maps IVs with correlated horizontal pleiotropy to cis‐associated genes and enriched pathways.	Correlated horizontal pleiotropy is detected and adjusted by using a Bayesian framework; provides robust estimates in cross‐population analyses.	Computationally intensive due to requiring correlation matrices; assumes that all IVs have potential uncorrelated horizontal pleiotropy, while only small number of IVs have correlated horizontal pleiotropy; requires dozens of IVs to detect and delineate correlated horizontal pleiotropy; accurate LD reference panel is required relies on the quality and resolution of GWAS.
MR‐Horse (Grant and Burgess 2024)	Uses horse‐shoe prior and allows adaptation of MR models to specific scenarios by adjusting the global shrinkage parameter.	Does not rely on the InSIDE assumption, models both correlated and uncorrelated pleiotropy, using a Bayesian framework; LD structure is incorporated in the likelihood.	Bayesian inference with LD matrices and multivariable models is computationally intensive; models are sensitive to prior specifications; relies on the quality and resolution of GWAS.

MR has been extended to allow prioritization of functionally pertinent genes at genetic loci reported by GWAS. If a genetic variant impacts expression levels of a given gene (i.e. the gene is subject to an eQTL), varying expression levels will be detected among individuals with different genotypes, conveying a concept similar to overexpression/suppression experiments. Subsequently, if gene expression levels also have an influence on a trait, there will be observable phenotypic differences depending on genotype (Zhu et al. 2016). MR can thus be used analogously to test for the causal effects of gene expression on complex traits. However, the statistical power of MR to detect these causal associations depends on the proportion of outcome variance explained by the exposure, the proportion of exposure variance explained by the IVs and the overall sample size (Brion et al. 2013). Given the polygenicity of most complex human traits, the variance of gene expression or phenotype explained by a single polymorphism (SNP) is usually small, such that sample sizes exceeding tens of thousands of individuals may be necessary to identify a gene's causal effect using MR. Summary data‐based MR (SMR) analysis, introduced in 2016, aggregated summary data from independent GWAS and eQTL studies in order to ascertain genes whose expression levels showed associations with a trait because of pleiotropy (Zhu et al. 2016). LD can also bias SMR, especially when gene expression is used as exposure: strong cis‐eQTLs located near their transcripts are usually correlated. Both pleiotropy and LD are violations of the third MR principle.

In standard MR, the effect of gene expression (*x*) on trait (*y*) can, in the absence of non‐genetic confounders, be expressed as a ratio of the IV (*z*) effects on x and y, respectively:

$$b_{xy}=b_{zy}/b_{zx,}$$

In the presence of latent non‐genetic confounding and under assumption of either causality or horizontal pleiotropy, the performance of MR and SMR was shown to be equivalent (Zhu et al. 2016). Statistical power of SMR analyses increases significantly when GWAS data derived independently of the eQTL dataset are employed (Zhu et al. 2016). Within SMR, the “heterogeneity in dependent instruments” (HEIDI) test has been developed to distinguish between pleiotropy and LD. The latter scenario occurs when the top cis‐eQTL is in LD with two discrete causal variants— one influencing gene expression and the other affecting trait variance (Zhu et al. 2016). Under pleiotropy, where a causal variant influences both gene expression levels and trait, all genetic markers in LD with a causal variant are expected to have same *b*_*xy*_ values as a causal variant. HEIDI assesses the heterogeneity of the SNPs' *b*_*xy*_ values in the same cis‐eQTL region. Importantly, SMR utilizes a univariable single instrument MR approach and therefore is not able to discriminate between pleiotropy and causality.

### 3.3 Transcriptome‐Wide Association Studies and Mendelian Randomization

TWAS, while not a formal type of IV analyses, shares conceptual similarities with MR in that both methods use genetic variation to investigate links between molecular traits and phenotypes. TWAS represent a family of statistical methods that has been widely used to find associations between complex traits and gene expression and search for causal gene candidates by combining GWAS and gene expression predictors from eQTL cohorts (de Leeuw et al. 2023). Although TWAS findings are often interpreted as evidence of a genetic relationship between phenotype and gene expression, this interpretation is not congruent with a null hypothesis that TWAS tests (de Leeuw et al. 2023). TWAS models test for a relationship between the phenotype and the *genetically predicted* component of gene expression, ignoring any uncertainty in the predicted gene expression. This means that, instead of directly testing the relationship between gene expression and phenotype, TWAS effectively assesses genetic association between the phenotype and SNPs local to the gene, irrespective of any genetic relationship with the gene expression (de Leeuw et al. 2023). Additionally, the extent to which standard errors vary across genes depends on gene size and strength of the association between gene expression and trait; all of these factors complicate post‐hoc corrections (de Leeuw et al. 2023). TWAS misapplication leads to inflated type 1 error rates and spurious associations that can render up to 40% of significant results invalid (de Leeuw et al. 2023)

Alternative methods that could fulfill the intended role of TWAS include local genetic correlation analyses, MR and colocalization (de Leeuw et al. 2023). All of these approaches require different assumptions but can be used to evaluate local genetic associations between gene expression and different traits (de Leeuw et al. 2023). Standard methods for one‐ or two‐sample MR allow for the uncertainty in the prediction of exposure, while more advanced MR methods (Table 2) allow one to detect and discount some of the pleiotropic IVs that would ordinarily be used to predict the gene expression. Nonetheless, it must be acknowledged that measurement error is a persistent issue not only in TWAS but across all causal inference methods (de Leeuw et al. 2023).

### 3.4 Relationships With Other Causal Inference Methods

The foundational principles and diverse applications of MR extend to other causal inference methods, facilitating more robust and comprehensive understanding of genetic causality. This section examines these synergistic relationships, detailing how MR can be applied to enhance colocalization and TWAS (Figure 2). The relationship between MR and network‐based approaches will be discussed later.

#### 3.4.1 Colocalization

MR and colocalization address different but related questions. While MR aims to determine the nature of relationship between exposure and outcome, colocalization assesses whether two traits are influenced by the same causal genetic variants – without needing to pre‐specify either of the traits as an exposure or outcome (Hukku et al. 2021). Although colocalization itself provides more robust mechanistic evidence of shared genetic etiology than simply observing statistical significance in the same genetic region for both traits (King et al. 2021), MR's causal framework can help to interpret colocalization results in a causal context. Integrative analyses combining MR and colocalization can highlight the genes involved in putative causal pathways (Liu et al. 2020). Colocalization identifies target genomic regions and causal variants, whereas MR estimates the overall causal effect of the candidate gene under an assumed direction of association (Zuber et al. 2022). Colocalization is often performed as a part of a broader causal inference pipeline and can be employed to validate IV assumptions prior to MR. By validating the shared genetic basis between GWAS loci and molecular traits, colocalization ensures integrity of genetic instruments. In cases where there is sufficient evidence that exposure and outcome are actually influenced by separate causal variants, it is unlikely that markers in that particular locus can be used as valid instruments for MR (Zuber et al. 2022). When both MR and colocalization provide concordant support, this significantly strengthens evidence confirming the causal role of the candidate gene in the trait development.

#### 3.4.2 Transcriptome‐Wide Association Studies

As mentioned, TWAS primarily evaluate the joint association between trait and SNPs in the region, rather than directly testing the causal role of gene expression (de Leeuw et al. 2023). Despite this distinction, MR principles have been adapted for TWAS applications, specifically by employing a probabilistic MR likelihood framework that unifies many TWAS and MR methods (Yuan et al. 2020). Using this approach, TWAS associations are modelled with multiple correlated SNPs that are later tested in an MR likelihood model explicitly accounting for horizontal pleiotropy (Yuan et al. 2020). Probabilistic MR methods use the burden test for horizontal pleiotropy modelling assumption and generalize MR‐Egger regression to multiple correlated IVs, thereby controlling for both correlated and uncorrelated pleiotropic effects (Yuan et al. 2020).

### 3.5 Recent Advancements in Mendelian Randomization

Multi‐instrument and multivariable MR has been successfully adapted to gene expression exposures by tailoring the inverse‐variance weighted method to GWAS and eQTL summary statistics, and assessing the multivariate causal effect of all genes at the given locus (Porcu et al. 2019a). This particular approach necessitates inclusion of all potential sources of measured pleiotropy in the model (Porcu et al. 2019a; Burgess and Thompson 2015). Other approaches that correct for pleiotropy have either entirely excluded pleiotropic IVs from models (Zhu et al. 2016; Verbanck et al. 2018; Zhu et al. 2018) or required exposures and outcomes to be derived from the same dataset (Berzuini et al. 2020a)—but both approaches risk loss of information and decrease in statistical power. Capturing all sources of pleiotropy is not always feasible, especially when the source originates from a gene in a different tissue or other unmeasured biomarkers or phenotypes (van der Graaf et al. 2020).

Several novel MR methods were developed in the last 5 years to address these methodological constraints. It has been demonstrated that a major source of pleiotropy in transcriptome‐wide MR comes from eQTLs of genes that are in LD with the primary IVs (van der Graaf et al. 2020). To address this issue, Mendelian randomization with Linkage Disequilibrium and Pleiotropy (MR‐link) employs eQTL summary statistics and individual‐level data for the exposure and outcome, respectively, and makes a casual inference about the effect of exposure while correcting for LD and unobserved pleiotropy. MR‐link does not require removal of pleotropic IVs and instead jointly analyses genetic markers that are in LD with exposure eQTLs (van der Graaf et al. 2020) (Table 2).

Traditional MR methods tackle correlated horizontal pleiotropy by assuming that it affects all IVs and estimating how much shared heritable factors contribute to IV‐outcome relationships (Morrison et al. 2020; Xue et al. 2021; Wang et al. 2021). The effective‐median‐based MR framework (EMIC) identifies false‐positives due to LD and/or correlation between IVs and uses multiple cis‐eQTLs to perform pleiotropy fine‐mapping (Jiang et al. 2022). In contrast to alternative methods, EMIC employed eigenvalue decomposition matrix estimated from ancestry matched reference panel, making it robust to the LD noise and discrepancies (Jiang et al. 2022) (Table 2).

The issue of IV invalidity due to correlated pleiotropy has also been tackled by introduction Bayesian framework to MR. Traditional frequentist methods that assess the violation of INSIDE assumption, rely on fulfilment of specific strict conditions and often suffer from inflated type I error rates (Morrison et al. 2020). Bayesian framework for MR was first introduced in a seminal work by Berzuini et al. who proposed a horseshoe prior to address the invalidity of IVs (Berzuini et al. 2020b). However, this particular method still relied on INSIDE assumption and required individual‐level data (Berzuini et al. 2020b), with subsequent improvements extending to summary data‐based Bayesian MR (Zhao et al. 2020; Bucur et al. 2020). Other Bayesian methods, such as Mendelian randomization with Correlated Horizontal Pleiotropy) (MR‐Corr^2^), tackled correlated pleiotropy by incorporating a correlation parameter in the model (Cheng et al. 2022a). Nonetheless, MR‐Corr^2^ was limited by assumption that pleiotropic effects were either entirely correlated or uncorrelated with IV effects on exposure (Cheng et al. 2022a). By contrast, Causal Analysis Using Summary Effect estimates (CAUSE) (Morrison et al. 2020)and Correlated horizontal pleiotropy Unravelling shared Etiology and confounding (MR‐CUE) (Cheng et al. 2022b), considered both correlated and uncorrelated pleiotropic effects, avoiding reverse causation and mitigating weak instruments bias (Cheng et al. 2022b). MR‐CUE mapped IVs with correlated horizontal pleiotropy to their respective cis‐associated genes and enriched pathways, informing shared genetic etiology between exposure and outcome (Cheng et al. 2022b). However, CAUSE and MR‐Cue that the association of IVs with exposure was either entirely due to confounders or solely because of a direct effect (Morrison et al. 2020; Cheng et al. 2022b) Recently developed MR‐Horse does not rely on INSIDE assumption, handles both correlated and uncorrelated pleiotropy, and retains low rates of type I error under wide range of different circumstances (Grant and Burgess 2024). MR‐Horse allows to adapt MR models to specific scenarios by adjusting the global shrinkage parameter (Grant and Burgess 2024).

### 3.6 Practical Scenarios

#### 3.6.1 Scenario 1—Mendelian Randomization Performing Well

Li et al. performed proteome‐wide MR analyses systematically investigating causal associations between 2490 circulating proteins 19 different cardiovascular diseases (CVDs) (Li et al. 2025). Instead of focusing on single protein‐disease associations, this compressive approach facilitated thorough exploration of protein‐mediated causal pathways across diverse CVD pathologies (Li et al. 2025). The analyses aggregated summary statistics from the largest published proteome dataset and meta‐analysis of GWAS on CVDs in European and Asian populations (Li et al. 2025). Integration of large well‐characterized datasets increased statistical power for detecting genuine causal associations (Li et al. 2025). Genetic instruments were derived separately for European and East‐Asian samples and analyzed with CVDs from corresponding ancestry GWAS (Li et al. 2025). This multi‐ancestry approach allowed to control for ancestry‐specific alleles, improving the accuracy of causal estimates and generalizability of study findings (Li et al. 2025). Bi‐directional MR was implemented to differentiate causal candidate proteins from reverse causality, a concern that was highlighted in previous MR investigations, that found a partial overlap between molecular causes and consequences of the disease (Li et al. 2025). MR identified 218 proteins that causally influenced the risk of one or several CVDs; of them 111 were novel associations (Li et al. 2025). Results of forward and reverse MR detected only 2 overlapping signals, which indicated that proteomic causes and consequences of CVDs were largely distinct (Li et al. 2025). Interestingly, among 15 protein biomarkers whose expression levels were influenced by CVD status, 5 were specific to East‐Asian populations which showcased the value of a multi‐ancestry MR approach (Li et al. 2025). Genes exclusive to East‐Asian ancestry among other things had cardioprotective effects on cardiomyocyte survival and stress‐induced angiogenesis (Hedhli et al. 2014) and were implicated in overweight‐related hypertension in Han‐Chinese (Zhu et al. 2021a). Candidate causal proteins for CVDs included drug targets, namely, ANGPTL3, ECE1 and PCSK9 inhibitors, that were already approved for CVD treatment or in development, thereby exemplifying successful application of MR in pharmacogenomics (Li et al. 2025). Among novel gene candidates, *BTN3A2* exhibited a relatively large causal effect on the risk of ischemic stroke; this association was consistent across different datasets and analyses with a crosstalk between immunomodulation and stroke emerging as a likely mechanism (Li et al. 2025). MR utilizing single‐cell RNA sequencing showed causal associations between *PAM* and *LPL* enrichment in cardiomyocytes and the risk of angina pectoris, ventricular arrythmia and peripheral artery diseases, respectively (Li et al. 2025). A study Li and colleagues showcased the potential of leveraging human genetics and MR for full‐scale novel drug discovery (Li et al. 2025).

#### 3.6.2 Scenario 2—Mendelian Randomization Producing Potentially Misleading Results

A study by Wu et al. investigated potential drug targets for MI by integrating European MI GWAS and blood plasma proteome data (Wu et al. 2023). Although the study identified *LPA* and *APOA5* as putative causal genes, there were several limitations pertaining to MR analysis (Wu et al. 2023). In MR, blood plasma cis‐pQTLs were used as exposure variables, and MI was the outcome (Wu et al. 2023). Associations between MI and blood plasma proteins represented by a single pQTL were analyzed using the Wald ratio test, whereas proteins with multiple IVs were assessed by the IWV test (Wu et al. 2023). Although both approaches were valid, the Wald‐ratio test in particular severely limited the options for MR sensitivity analyses, such as MR‐Egger regression or heterogeneity test (Wu et al. 2023). Conversely, the IVW test required that the sum of horizontal pleiotropic effects across all IVs was equal to zero, assuming that strength of IVs was also not related to pleiotropy (Burgess et al. 2013). The study did not formally test for pleiotropy, which meant that one of the core MR assumptions ‐ that IV affects the outcome through exposure‐ could not be verified (Wu et al. 2023). Even though cis‐pQTLs were believed to be less vulnerable to pleiotropy compared to trans‐pQTLs (Zheng et al. 2020), they still could affect neighboring genes, as seen in studies of gene expression (Zheng et al. 2020). Additionally, MR analyses, where genetic instruments were restricted to a single region, not only exhibited reduced statistical power but also inflated type I error rates (van der Graaf et al. 2025). Inclusion of non‐pleiotropic trans‐pQTL in MR analyses could potentially improve the consistency of protein‐trait associations by increasing the variance explained by target proteins (Zheng et al. 2020). Moreover, this approach would ensure that casual estimates were not reliant on a single genetic locus and additional sensitivity analyses could be performed (Zheng et al. 2020). By instrumenting blood plasma protein levels, Wu et al. selected a tissue that might not have been relevant for the development of MI (Wu et al. 2023). Mismatches between tissues where protein exposures were measured and disease‐relevant pQTLs can lead to spurious associations (Porcu et al. 2019b). Overall, the investigation by Wu and colleagues exemplifies how availability of rich and diverse QTL data is currently one of the major obstacles in causal inference research.

## 4 Network‐Based Approaches

### 4.1 Historical Context and General Use Cases

Although MR has gained popularity as a method for causal inference in recent years, limited options exist for its use in the systemic interrogation of omic data (Yazdani et al. 2022). Indeed, its hypothesis‐driven approach does not consider complex relationships and interactions between diverse types of molecules that may contribute to disease development; even when multiple exposures are considered (as in multivariable MR), the goal is to assess individual causes (Yazdani et al. 2022). Furthermore, limited prior knowledge about the relationships between different genetic/environmental risk factors makes it challenging to delineate explanatory, mediating and response variables using MR.

**Figure 4:**
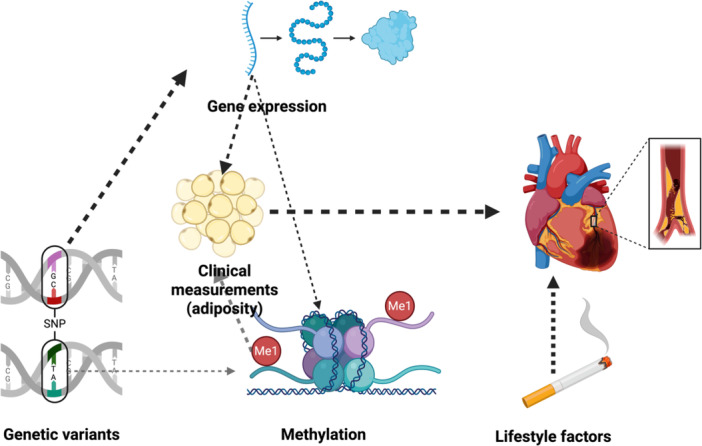
Schematic representation of network‐based approaches for causal inference. Genetic variants can be used as anchors in causal networks to help orient the edges (dashed lines) between different nodes, here represented by different risk factors. Figure was produced in Biorender.

In contrast to MR, network‐based analyses can be used to systematically integrate genomic and multi‐omics data, attempting to infer causal structures (Liu et al. 2009; Mezlini and Goldenberg 2017); as such, these approaches are better suited for multi‐omics data typically characterized by high dimensionality, interdependency between layers and pleiotropy. The origins of network‐based causal inference approaches can be traced to the 20th century when Sewall Wright laid foundations for structural equation modelling aimed at describing relationships between different variables (Niles 1922). So‐called causal networks (CNs) provide structured discovery‐based analyses of complex data where each entity, represented by nodes in the network, can be an explanatory variable, mediator or a response (Yazdani et al. 2022). While MR employs genetic variants as IVs to isolate causal effects in observational studies, network‐based approaches use directed acyclic graphs to model conditional dependencies among variables (Figure 4) (Howey et al. 2020).

### 4.2 Core Approach

It is important to remember that MR principles are embedded in the causal framework of network‐based models. One of the advantages of network‐based approaches over MR is that they can handle large datasets such as omics measurements. MR approaches can be used to help build CNs depending on the type of available omics data (Yazdani et al. 2022). Associations identified in MR can be incorporated as “genetic anchors” within the causal network, guiding and constraining the network structure (Howey et al. 2020). This way MR helps improve the accuracy and biological plausibility of inferred relationships by grounding them in strong causal evidence that is less affected by confounding compared to purely observational associations (Yazdani et al. 2022). QTLs can be included as IVs for genes whose levels are coded by one or two genetic markers, whereas polygenic factors may explain a larger proportion of phenotypic variation and show stronger associations with other explanatory variables in the model (Yazdani et al. 2022). The polygenic approach provides additional benefits over MR, such as reduction in spurious and highly sensitive estimates as well as enhanced statistical power by assigning multiple IVs to explanatory variables (Yazdani et al. 2022). Bayesian networks (BN) are probabilistic graphical models signifying a set of entities and conditional relationships between them as directed acyclic graphs (DAGs) (Puga et al. 2015). Independent variables and probabilistic dependencies in the DAG are represented by nodes and edges (Puga et al. 2015). The conditional probability distribution of each node quantifies the effects of its parent nodes, allowing the interpretation of edges as causal associations between exposure and outcome (Puga et al. 2015). BNs with genetic anchors can infer causal relationships between multiple genetic and non‐genetic risk factors at the same time, revealing complex causal networks, particularly in the context of horizontal pleiotropy (Howey et al. 2020).

### 4.3 Relationships With Other Causal Inference Methods

Network‐based analyses provide a causal inference framework that can complement and improve utility and interpretability of colocalization, Mendelian randomization and TWAS analyses by integrating their findings into a cohesive, systemic view of biological processes (Figure 2).

#### 4.3.1 Colocalization

Network‐based causal inference methods can foster synthesis of colocalization evidence across multiple omic layers by jointly modelling gene products and improving causal variant resolution (Okamoto et al. 2025). This multi‐layered approach helps to discern whether an observed colocalization signal truly reflects shared causality or is confounded by other regulatory mechanisms. Causal networks can link genetic variants through various molecular intermediates, ultimately revealing a more complete mechanistic milieu (Agamah et al. 2022). Placing colocalization signals in a broader etiological context shows how shared genetic markers influence biological systems through involvement with upstream regulators, downstream effectors and other regulatory mechanisms. When colocalization points to a shared genetic basis between two nodes in the network, the network structure can inform interactions with other molecules or pathways implicated in shared genetic mechanisms (Barrio‐Hernandez et al. 2023).

#### 4.3.2 Mendelian Randomization

Foundational MR principles can be embedded in the construction of causal networks by implementing methodological advancements that extend the core logic of using genetic variants as IVs (Yazdani et al. 2022) Many of network‐based causal inference methods use a two‐step approach, whereby they initially draw a totality of causal influences among traits and later refine these general connections by pinpointing direct causal relationships in the network (Lin et al. 2023). In this initial step, extended MR methods are employed to infer all possible causal connections without distinguishing between immediate effects and intermediate associations (Lin et al. 2023).

Causal networks expand MR principles to encompass multiple exposures and outcomes, thereby moving beyond the hypothesis‐driven paradigm of MR (Yazdani et al. 2022). The discovery‐based approach implemented in network‐based causal inference enables mapping of causal pathways and cascades that include numerous complex interactions (Lin et al. 2023). Traditional MR mediation analyses typically focus on one mediator in a specific causal pathway, and while extensions exist (Yang et al. 2024a), they can become unwieldy when multiple mediators are considered. In contrast to MR, causal networks assess the overall architecture of mediation rather than isolated mediation paths by quantifying contributions of multiple intermediate variables and indirect effects (Lin et al. 2023). Network‐based causal inference methods are able to isolate direct effects by applying graph deconvolution to the network of total effects (Lin et al. 2023; Feizi et al. 2013). Additionally, by analyzing conditional dependencies and the overall structure of the network, network‐based causal inference methods can detect and differentiate various forms of genetic confounding (Yazdani et al. 2022; Amar et al. 2021), whereas MR often operates under simplified assumptions and requires additional sensitivity analyses (Carter et al. 2021).

#### 4.3.3 Transcriptome‐Wide Association Studies

Although TWAS can inform on the relationship between gene expression and phenotype at the genomic region level, they overlook complex gene‐gene interactions and interdependencies and are unable to distinguish direct causal effects from downstream effects within the same pathway (Subirana‐Granés et al. 2025; Pividori et al. 2023). Additionally, as mentioned previously, TWAS findings can be confounded by factors such as LD and pleiotropy which may result in false‐positive associations (de Leeuw et al. 2023). Network‐based approaches offer structural context for TWAS‐reported genes and refine their prioritization by assessing their position and connectivity within a wider biological network (Kaushal and Singh 2020; Jin et al. 2022). This enables a more accurate detection of causal genes by embedding them in the framework that considers their interdependencies (Jin et al. 2022). When a broader regulatory topology and interactions between genes are considered, genetic confounding arising from pleiotropy and LD can also be minimised (Sun et al. 2024; Zhu et al. 2021b). Additionally, network‐based causal inference methods can be used to forecast genetic contribution of gene expression by integrating distal genetic variants that operate through gene regulatory networks, consistent with omnigenic model of trait inheritance (Mohammad and Michoel 2024), Using this approach, Bayesian networks capture causal and coexpression relationships between genes and improve gene expression prediction accuracy which is crucial for TWAS (Mohammad and Michoel 2024).

### 4.4 Recent Advancements in Network‐Based Methods

BNs address some of MR's shortcomings, particularly when handling high‐dimensional datasets with multiple genetic predictors and potential risk factors (Howey et al. 2020). The advantages of BNs have been demonstrated in situations with widespread pleiotropy, where this method outperformed bi‐directional MR in terms of type I error control and statistical power (Howey et al. 2020). BNs have been successfully used to scan for disease‐associated genes across a wide range of complex disorders like breast cancer, bone metastasis (Park et al. 2018) and Alzheimer's (Sherif et al. 2015). For gene regulatory network inference, hybrid methods integrating differential equations with dynamic Bayesian networks have been developed. The Differential Equation based Local Dynamic Bayesian Network (DELDBN) algorithm demonstrated a notable enhancement in accuracy and scalability when applied to yeast and human cell data, successfully reconstructing known interactions around the *BRCA1* gene (Li et al. 2011). In analyses of protein sequences, feature extraction methods using Bayesian networks and Dirichlet distributions have been employed for causal inference (Liu et al. 2009). Additionally, recent developments like the Causal Inference Using Composition of Transactions (CICT) method has shown promising results in uncovering gene regulatory networks from single‐cell RNA‐seq data, outperforming existing network inference methods (Shojaee and Huang 2023). For situations with scarce data, knowledge transfer techniques that utilize data from other sources have been suggested (Rodríguez‐López and Sucar 2022). To ensure accurate and reliable BN analysis, missing information can be imputed using the nearest neighbor approach (Howey et al. 2021) or survival tree analysis (Rancoita et al. 2016).

Violations of the core MR assumption result in unstable networks. When directional anchors are not included in BNs, several issues can arise, including poor accuracy, confounding and pleiotropy (Xue et al. 2021) all of which contribute to less stable networks. The assessment of network stability involves an array of methodologies. In bootstrapping, the variability of estimated network parameters is evaluated by resampling of the data with subsequent replacement (Dikopoulou 2021). Sensitivity analyses test how systemic variations in inputs and parameters of the models affect network stability (Yazdani et al. 2022). The performance of network‐based approaches under different conditions, such as sample size and data noisines,s can be assessed in simulation studies (Yazdani et al. 2022; Dikopoulou 2021)

BNs augmented with principles of MR provide a valuable complementary approach to existing causal inference methods, particularly in the context of modern high‐dimensional omics (Howey et al. 2020; Evans and Davey Smith 2015). Ongoing research efforts continue to improve BNs scalability and applicability to large‐scale genomics (Ji et al. 2015; Larjo et al. 2013).

### 4.5 Practical Scenarios

#### 4.5.1 Scenario 1—Network‐Based Approaches Performing Well

A study conducted by Zeng et al. exemplifies how network‐based causal inference analyses can be successfully utilized to uncover genetic variance and causal mechanisms underlying CAD (Zeng et al. 2019). This particular investigation sought to assess heritability contributions of SNPs associated with gene‐expression (e‐SNPs) in CAD‐related gene‐regulatory networks. Seven vascular and metabolic eQTL datasets from patients with CAD were used to detect e‐SNPs and build co‐expression networks (Zeng et al. 2019). Prior information from co‐expression network eQTLs was utilized in the Bayesian algorithm that inferred gene regulatory networks (GRNs) with causal links to CAD (Zeng et al. 2019). This approach allowed the detection not only of disease‐associated putatively causal genes but also of entire etiological pathways. By incorporating the genetics of gene expression, causal networks revealed directionality of gene interactions and identified hub genes that functioned as key network drivers, regulating downstream genes (Zeng et al. 2019). The causal role of these “hub” genes was further supported by in vitro models that demonstrated changed activity of entire GRNs active in CAD and impact on CAD phenotype (Zeng et al. 2019). Importantly, among all analyzed tissues, fat and arterial wall exhibited the strongest influence of the risk of CAD (Zeng et al. 2019). GRNs involved, among known CAD risk factors, RNA metabolism, DNA binding and blood coagulation mechanisms. Moreover, Zang and colleagues determined that e‐SNPs underpinning gene regulatory networks explained an additional 10% of CAD heritability not explained by GWAS (Zeng et al. 2019). Twenty‐eight GRNs were replicated in an independent dataset which underscored the robustness of the study findings (Zeng et al. 2019).

#### 4.5.2 Scenario 2—Network‐Based Approaches Producing Potentially Misleading Results

Bowles and colleagues employed the BN approach in the analyses of modifiable and non‐modifiable cardiovascular risk factors (Bowles et al. 2024). The study determined that beyond traditional risk factors, such as BMI and physical activity, ethnicity and exposure to heavy metals were the strongest drivers of CVD Blood levels of heavy metals mediated the relationship between ethnicity and cardiovascular disease (Bowles et al. 2024). Although the study demonstrated the utility of using BNs for understanding complex interactions between different variables influencing CVD risk, there were several notable limitations that could affect the accuracy of the results and interpretation of causal associations (Bowles et al. 2024). For example, ascertaining directions of the edges in the network relied on specific assumptions. The edge was included in the network if it showed up between the two nodes in at least 51% of bootstrapped networks (Bowles et al. 2024). Consequently, this approach assumed that the relationships between variables were stable under bootstrapping conditions (Bowles et al. 2024). Despite statistical support provided by the BN model, inferred causal directions might not fully capture true biological causality without additional sensitivity analyses evaluating model assumptions (Bowles et al. 2024). Additionally, even with the implementation of BNs, concurrent study design meant that the possibility of reverse causation and bidirectional relationships could not be completely ruled out (Bowles et al. 2024). Longitudinal, experimental and quasi‐experimental methods could be adopted as robust alternatives, ensuring the integrity of BN modelling.

## 5 Future Perspectives

Integration of methods from the causal inference literature into genomic studies has shown great promise in unravelling complex biological relationships, with techniques like MR, and colocalization as well as network‐based approaches identifying potential causal genes and furthering the understanding of genetic architecture of complex traits and diseases (van der Graaf et al. 2020; Howey et al. 2020; Zhao et al. 2024; Yang et al. 2024b). However, as mentioned, these methods face challenges in the presence of factors such as pleiotropy, LD, and genetic confounding. TWAS and other eQTL‐based methods, in particular, may prioritize multiple genes at a locus, some of which may be non‐causal due to shared eQTLs (Wainberg et al. 2019). To address these issues, novel approaches like MR‐link and causal‐TWAS (cTWAS) have been developed, which account for unobserved pleiotropy and genetic confounding (van der Graaf et al. 2020; Zhao et al. 2024).

Looking ahead, the integration of multiple data types and the development of more sophisticated statistical frameworks will be crucial. The advent of methods combining colocalization and MR analysis for loci with allelic heterogeneity represents a promising step in this direction (Zhu et al. 2021c). Additionally, the incorporation of tissue‐specific eQTL data and the consideration of context‐dependent effects will be important for improving the accuracy of causal inference (Wainberg et al. 2019).

## Conflicts of Interest

The authors declare no conflicts of interest.

## References

[gepi70037-bib-0001] Agamah, F. E. , J. R. Bayjanov , and A. Niehues , et al. 2022 November 14. “Computational Approaches for Network‐Based Integrative Multi‐Omics Analysis.” Frontiers in Molecular Biosciences 9: 967205. https://www.frontiersin.org/journals/molecular-biosciences/articles/10.3389/fmolb.2022.967205/full. 36452456 10.3389/fmolb.2022.967205 PMC9703081

[gepi70037-bib-0002] Al‐Barghouthi, B. M. , W. T. Rosenow , K. P. Du , et al. 2022 November 23. “Transcriptome‐Wide Association Study and eQLT Colocalization Identify Potentially Causal Genes Responsible for Human Bone Mineral Density Gwas Associations.” eLife 11: e77285. 36416764 10.7554/eLife.77285 PMC9683789

[gepi70037-bib-0003] Amar, D. , N. Sinnott‐Armstrong , E. A. Ashley , and M. A. Rivas . 2021 January 13. “Graphical Analysis for Phenome‐Wide Causal Discovery in Genotyped Population‐Scale Biobanks.” Nature Communications 12, no. 1: 350. 10.1038/s41467-020-20516-2 PMC7806647 33441555

[gepi70037-bib-0004] Arvanitis, M. , K. Tayeb , B. J. Strober , and A. Battle . 2022a February 3. “Redefining Tissue Specificity of Genetic Regulation of Gene Expression in the Presence of Allelic Heterogeneity.” American Journal of Human Genetics 109, no. 2: 223–239. 35085493 10.1016/j.ajhg.2022.01.002 PMC8874223

[gepi70037-bib-0005] Arvanitis, M. , K. Tayeb , B. J. Strober , and A. Battle . 2022b February 3. “Redefining Tissue Specificity of Genetic Regulation of Gene Expression in the Presence of Allelic Heterogeneity.” American Journal of Human Genetics 109, no. 2: 223–239. 35085493 10.1016/j.ajhg.2022.01.002 PMC8874223

[gepi70037-bib-0006] Aygün, N. , D. Liang , W. L. Crouse , G. R. Keele , M. I. Love , and J. L. Stein . 2023 May 30. “Inferring Cell‐Type‐Specific Causal Gene Regulatory Networks During Human Neurogenesis.” Genome Biology 24, no. 1: 130. 37254169 10.1186/s13059-023-02959-0 PMC10230710

[gepi70037-bib-0007] Barrio‐Hernandez, I. , J. Schwartzentruber , A. Shrivastava , et al. 2023 March “Network Expansion of Genetic Associations Defines a Pleiotropy Map of Human Cell Biology.” Nature Genetics 55, no. 3: 389–398. 36823319 10.1038/s41588-023-01327-9 PMC10011132

[gepi70037-bib-0008] Berzuini, C. , H. Guo , S. Burgess , and L. Bernardinelli . 2020a January 1. “A Bayesian Approach to Mendelian Randomization With Multiple Pleiotropic Variants.” Biostatistics 21, no. 1: 86–101. 30084873 10.1093/biostatistics/kxy027 PMC6920542

[gepi70037-bib-0009] Berzuini, C. , H. Guo , S. Burgess , and L. Bernardinelli . 2020b January 1. “A Bayesian Approach to Mendelian Randomization With Multiple Pleiotropic Variants.” Biostatistics 21, no. 1: 86–101. 30084873 10.1093/biostatistics/kxy027 PMC6920542

[gepi70037-bib-0010] Bowden, J. , G. Davey Smith , and S. Burgess . 2015 April 1. “Mendelian Randomization With Invalid Instruments: Effect Estimation and Bias Detection Through Egger Regression.” International Journal of Epidemiology 44, no. 2: 512–525. 26050253 10.1093/ije/dyv080 PMC4469799

[gepi70037-bib-0011] Bowden, J. , G. Davey Smith , P. C. Haycock , and S. Burgess . 2016b. “Consistent Estimation in Mendelian Randomization With Some Invalid Instruments Using a Weighted Median Estimator.” Genetic Epidemiology 40, no. 4: 304–314. 27061298 10.1002/gepi.21965 PMC4849733

[gepi70037-bib-0012] Bowden, J. , M. F. Del Greco , C. Minelli , G. Davey Smith , N. A. Sheehan , and J. R. Thompson . 2016a December 1. “Assessing the Suitability of Summary Data for Two‐Sample Mendelian Randomization Analyses Using Mr‐Egger Regression: The Role of the I2 Statistic.” International Journal of Epidemiology 45, no. 6: 1961–1974. 27616674 10.1093/ije/dyw220 PMC5446088

[gepi70037-bib-0013] Bowden, J. , M. F. Del Greco , C. Minelli , et al. 2019 June 1. “Improving the Accuracy of Two‐Sample Summary‐Data Mendelian Randomization: Moving Beyond the Nome Assumption.” International Journal of Epidemiology 48, no. 3: 728–742. 30561657 10.1093/ije/dyy258 PMC6659376

[gepi70037-bib-0014] Bowles, N. P. , Y. He , Y. H. Huang , E. C. Stecker , A. Seixas , and S. S. Thosar . 2024 June 10. “Cardiovascular Disease Risk: It Is Complicated, but Race and Ethnicity Are Key, a Bayesian Network Analysis.” Frontiers in Public Health 12: 1364730. https://www.frontiersin.org/journals/public-health/articles/10.3389/fpubh.2024.1364730/full. 38915752 10.3389/fpubh.2024.1364730 PMC11194318

[gepi70037-bib-0015] Brion, M. J. A. , K. Shakhbazov , and P. M. Visscher . 2013 October 1. “Calculating Statistical Power in Mendelian Randomization Studies.” International Journal of Epidemiology 42, no. 5: 1497–1501. 24159078 10.1093/ije/dyt179 PMC3807619

[gepi70037-bib-0016] Bucur, I. G. , T. Claassen , and T. Heskes . 2020 April 1. “Inferring the Direction of a Causal Link and Estimating Its Effect via a Bayesian Mendelian Randomization Approach.” Statistical Methods in Medical Research 29, no. 4: 1081–1111. 31146640 10.1177/0962280219851817 PMC7221461

[gepi70037-bib-0017] Burgess, S. , A. Butterworth , and S. G. Thompson . 2013. “Mendelian Randomization Analysis With Multiple Genetic Variants Using Summarized Data.” Genetic Epidemiology 37, no. 7: 658–665. 24114802 10.1002/gepi.21758 PMC4377079

[gepi70037-bib-0018] Burgess, S. , D. S. Small , and S. G. Thompson . 2017 October 1. “A Review of Instrumental Variable Estimators for Mendelian Randomization.” Statistical Methods in Medical Research 26, no. 5: 2333–2355. 26282889 10.1177/0962280215597579 PMC5642006

[gepi70037-bib-0019] Burgess, S. , and S. G. Thompson . 2015 Feb 15. “Multivariable Mendelian Randomization: The Use of Pleiotropic Genetic Variants to Estimate Causal Effects.” American Journal of Epidemiology 181, no. 4: 251–260. 25632051 10.1093/aje/kwu283 PMC4325677

[gepi70037-bib-0020] Carter, A. R. , E. Sanderson , G. Hammerton , et al. 2021 May 1. “Mendelian Randomisation for Mediation Analysis: Current Methods and Challenges for Implementation.” European Journal of Epidemiology 36, no. 5: 465–478. 33961203 10.1007/s10654-021-00757-1 PMC8159796

[gepi70037-bib-0021] Chen, B. Y. , W. P. Bone , K. Lorenz , M. Levin , M. D. Ritchie , and B. F. Voight . 2022 September 15. “ColocQuiaL: A QTL‐GWAS Colocalization Pipeline.” Bioinformatics 38, no. 18: 4409–4411. 35894642 10.1093/bioinformatics/btac512 PMC9477517

[gepi70037-bib-0022] Chen, L. G. , J. D. Tubbs , Z. Liu , T. Q. Thach , and P. C. Sham . 2024 June. “Mendelian Randomization: Causal Inference Leveraging Genetic Data.” Psychological Medicine 54, no. 8: 1461–1474. 38639006 10.1017/S0033291724000321

[gepi70037-bib-0023] Cheng, Q. , T. Qiu , X. Chai , et al. 2022a January 1. “MR‐Corr2: A Two‐Sample Mendelian Randomization Method That Accounts for Correlated Horizontal Pleiotropy Using Correlated Instrumental Variants.” Bioinforma Oxf Engl 38, no. 2: 303–310. 10.1093/bioinformatics/btab646 34499127

[gepi70037-bib-0024] Cheng, Q. , X. Zhang , L. S. Chen , and J. Liu . 2022b October 30. “Mendelian Randomization Accounting for Complex Correlated Horizontal Pleiotropy While Elucidating Shared Genetic Etiology.” Nature Communications 13, no. 1: 6490. 10.1038/s41467-022-34164-1 PMC9618026 36310177

[gepi70037-bib-0025] Costanzo, M. C. , L. W. Harris , Y. Ji , A. McMahon , N. P. Burtt , and J. Flannick . 2025 July. “Realizing the Promise of Genome‐Wide Association Studies for Effector Gene Prediction.” Nature Genetics 57, no. 7: 1578–1587. 40442285 10.1038/s41588-025-02210-5 PMC12286715

[gepi70037-bib-0026] Cui, M. , C. Cheng , and L. Zhang . 2022 Nov. “High‐Throughput Proteomics: A Methodological Mini‐Review.” Laboratory Investigation 102, no. 11: 1170–1181. 35922478 10.1038/s41374-022-00830-7 PMC9362039

[gepi70037-bib-0027] Dikopoulou, Z. 2021. “Network Analysis, Accuracy and Stability of the Job‐Satisfaction Structures.” In Modeling and Simulating Complex Business Perceptions: Using Graphical Models and Fuzzy Cognitive Maps [Internet], edited by Z. Dikopoulou , 85–118. Springer International Publishing. 10.1007/978-3-030-81496-0_5.

[gepi70037-bib-0028] Evans, D. M. , and G. Davey Smith . 2015 August 24. “Mendelian Randomization: New Applications in the Coming Age of Hypothesis‐Free Causality.” Annual Review of Genomics and Human Genetics 16, no. Volume 16, 2015: 327–350. 10.1146/annurev-genom-090314-050016 25939054

[gepi70037-bib-0029] Feizi, S. , D. Marbach , M. Médard , and M. Kellis . 2013 August. “Network Deconvolution as a General Method to Distinguish Direct Dependencies in Networks.” Nature Biotechnology 31, no. 8: 726–733. 10.1038/nbt.2635 PMC3773370 23851448

[gepi70037-bib-0030] Foley, C. N. , J. R. Staley , P. G. Breen , et al. 2021 February 3. “A Fast and Efficient Colocalization Algorithm for Identifying Shared Genetic Risk Factors Across Multiple Traits.” Nature Communications 12, no. 1: 764. 10.1038/s41467-020-20885-8 PMC7858636 33536417

[gepi70037-bib-0031] Franceschini, N. , C. Giambartolomei , P. S. de Vries , et al. 2018 December 3. “Gwas and Colocalization Analyses Implicate Carotid Intima‐Media Thickness and Carotid Plaque Loci in Cardiovascular Outcomes.” Nature Communications 9, no. 1: 5141. 10.1038/s41467-018-07340-5 PMC6277418 30510157

[gepi70037-bib-0032] Giambartolomei, C. , D. Vukcevic , E. E. Schadt , et al. 2014 May 15. “Bayesian Test for Colocalisation Between Pairs of Genetic Association Studies Using Summary Statistics.” PLoS Genetics 10, no. 5: e1004383. 24830394 10.1371/journal.pgen.1004383 PMC4022491

[gepi70037-bib-0033] van der Graaf, A. , A. Claringbould , A. Rimbert , et al. 2020 October 1. “Mendelian Randomization While Jointly Modeling Cis Genetics Identifies Causal Relationships Between Gene Expression and Lipids.” Nature Communications 11, no. 1: 4930. 10.1038/s41467-020-18716-x PMC7530717 33004804

[gepi70037-bib-0034] van der Graaf, A. , R. Warmerdam , C. Auwerx , et al. 2025 July 3. “MR‐link‐2: Pleiotropy Robust Cis Mendelian Randomization Validated in Three Independent Reference Datasets of Causality.” Nature Communications 16, no. 1: 6112. 10.1038/s41467-025-60868-1 PMC12229666 40610416

[gepi70037-bib-0035] Grant, A. J. , and S. Burgess . 2024 January 4. “A Bayesian Approach to Mendelian Randomization Using Summary Statistics in the Univariable and Multivariable Settings With Correlated Pleiotropy.” The American Journal of Human Genetics 111, no. 1: 165–180. 38181732 10.1016/j.ajhg.2023.12.002 PMC10806746

[gepi70037-bib-0036] Gray, R. , and K. Wheatley . 1991. “How to Avoid Bias When Comparing Bone Marrow Transplantation With Chemotherapy.” Bone Marrow Transplantation 7 Suppl 3: 9–12. 1855097

[gepi70037-bib-0037] Hedhli, N. , A. Kalinowski , and K. Russell . 2014. “Cardiovascular Effects of Neuregulin‐1/ErbB Signaling: Role in Vascular Signaling and Angiogenesis.” Current Pharmaceutical Design 20, no. 30: 4899–4905. 24283954 10.2174/1381612819666131125151058

[gepi70037-bib-0038] Hingorani, A. , and S. Humphries . 2005 December 3. “Nature's Randomised Trials.” Lancet 366, no. 9501: 1906–1908. 16325682 10.1016/S0140-6736(05)67767-7

[gepi70037-bib-0039] Hormozdiari, F. , M. van de Bunt , A. V. Segrè , et al. 2016 December 1. “Colocalization of Gwas and eQTL Signals Detects Target Genes.” American Journal of Human Genetics 99, no. 6: 1245–1260. 27866706 10.1016/j.ajhg.2016.10.003 PMC5142122

[gepi70037-bib-0040] Howey, R. , A. D. Clark , N. Naamane , L. N. Reynard , A. G. Pratt , and H. J. Cordell . 2021 September 29. “A Bayesian Network Approach Incorporating Imputation of Missing Data Enables Exploratory Analysis of Complex Causal Biological Relationships.” PLoS Genetics 17, no. 9: e1009811. 34587167 10.1371/journal.pgen.1009811 PMC8504979

[gepi70037-bib-0041] Howey, R. , S. Y. Shin , C. Relton , G. Davey Smith , and H. J. Cordell . 2020 March 2. “Bayesian Network Analysis Incorporating Genetic Anchors Complements Conventional Mendelian Randomization Approaches for Exploratory Analysis of Causal Relationships in Complex Data.” PLoS Genetics 16, no. 3: e1008198. 32119656 10.1371/journal.pgen.1008198 PMC7067488

[gepi70037-bib-0042] Hukku, A. , M. Pividori , F. Luca , R. Pique‐Regi , H. K. Im , and X. Wen . 2021 January 7. “Probabilistic Colocalization of Genetic Variants From Complex and Molecular Traits: Promise and Limitations.” American Journal of Human Genetics 108, no. 1: 25–35. 33308443 10.1016/j.ajhg.2020.11.012 PMC7820626

[gepi70037-bib-0043] Ji, Z. , Q. Xia , and G. Meng . 2015. “A Review of Parameter Learning Methods in Bayesian Network.” In Advanced Intelligent Computing Theories and Applications, edited by D. S. Huang and K. Han , 3–12. Springer International Publishing.

[gepi70037-bib-0044] Jiang, L. , L. Miao , G. Yi , et al. 2022 May 5. “Powerful and Robust Inference of Complex Phenotypes' Causal Genes With Dependent Expression Quantitative Loci by a Median‐Based Mendelian Randomization.” American Journal of Human Genetics 109, no. 5: 838–856. 35460606 10.1016/j.ajhg.2022.04.004 PMC9118119

[gepi70037-bib-0045] Jin, X. , L. Zhang , J. Ji , T. Ju , J. Zhao , and Z. Yuan . 2022 August 6. “Network Regression Analysis in Transcriptome‐Wide Association Studies.” BMC Genomics 23, no. 1: 562. 35933330 10.1186/s12864-022-08809-w PMC9356418

[gepi70037-bib-0046] Kaushal, P. , and S. Singh . 2020 August 6. “Network‐Based Disease Gene Prioritization Based on Protein–Protein Interaction Networks.” Network Modeling Analysis in Health Informatics and Bioinformatics 9, no. 1: 55.

[gepi70037-bib-0047] Khasawneh, L. Q. , Z. N. Al‐Mahayri , and B. R. Ali . 2022 June 1. “Mendelian Randomization in Pharmacogenomics: The Unforeseen Potentials.” Biomedicine & Pharmacotherapy 150: 112952. 35429744 10.1016/j.biopha.2022.112952

[gepi70037-bib-0048] King, E. A. , F. Dunbar , J. W. Davis , and J. F. Degner . 2021 May 17. “Estimating Colocalization Probability From Limited Summary Statistics.” BMC Bioinformatics 22, no. 1: 254. 34000989 10.1186/s12859-021-04170-z PMC8130535

[gepi70037-bib-0049] Larjo, A. , I. Shmulevich , and H. Lähdesmäki . 2013. “Structure Learning for Bayesian Networks as Models of Biological Networks.” In Data Mining for Systems Biology: Methods and Protocols [Internet], edited by H. Mamitsuka , C. DeLisi , and M. Kanehisa , 35–45. Humana Press. 10.1007/978-1-62703-107-3_4. 23192539

[gepi70037-bib-0050] de Leeuw, C. , J. Savage , I. G. Bucur , T. Heskes , and D. Posthuma . 2022a June. “Understanding the Assumptions Underlying Mendelian Randomization.” European Journal of Human Genetics 30, no. 6: 653–660. 35082398 10.1038/s41431-022-01038-5 PMC9177700

[gepi70037-bib-0051] de Leeuw, C. , J. Savage , I. G. Bucur , T. Heskes , and D. Posthuma . 2022b June. “Understanding the Assumptions Underlying Mendelian Randomization.” European Journal of Human Genetics 30, no. 6: 653–660. 35082398 10.1038/s41431-022-01038-5 PMC9177700

[gepi70037-bib-0052] de Leeuw, C. , J. Werme , J. E. Savage , W. J. Peyrot , and D. Posthuma . 2023 September 7. “On the Interpretation of Transcriptome‐Wide Association Studies.” PLoS Genetics 19, no. 9: e1010921. 37676898 10.1371/journal.pgen.1010921 PMC10508613

[gepi70037-bib-0053] Li, C. , N. De Jay , S. S. Zhang , et al. 2025. “Proteome‐Wide Mendelian Randomization Identifies Candidate Causal Proteins for Cardiovascular Diseases.” Advanced Genetics 6, no. 2: 2500003. 40657554 10.1002/ggn2.202500003 PMC12245532

[gepi70037-bib-0054] Li, Z. , J. Liu , P. Li , and A. Krishnan . 2011 August 4. “Large‐Scale Dynamic Gene Regulatory Network Inference Combining Differential Equation Models With Local Dynamic Bayesian Network Analysis.” Bioinformatics 27, no. 19: 2686–2691. https://discovery.researcher.life/article/large-scale-dynamic-gene-regulatory-network-inference-combining-differential-equation-models-with-local-dynamic-bayesian-network-analysis/61618f9f604f36bf929df1c55e7f5aa5. 21816876 10.1093/bioinformatics/btr454

[gepi70037-bib-0055] Lin, Z. , H. Xue , and W. Pan . 2023 May 18. “Combining Mendelian Randomization and Network Deconvolution for Inference of Causal Networks With Gwas Summary Data.” PLoS Genetics 19, no. 5: e1010762. 37200398 10.1371/journal.pgen.1010762 PMC10231771

[gepi70037-bib-0056] Liu, J. , M. Deng , and M. Qian . 2009. “Feature‐Based Causal Structure Discovery in Protein and Gene Expression Data With Bayesian Network.” 2009 Fifth International Conference on Natural Computation 1: 144–148. https://ieeexplore.ieee.org/document/5362831.

[gepi70037-bib-0057] Liu, Q. , J. Pan , C. Berzuini , M. K. Rutter , and H. Guo . 2020 May 4. “Integrative Analysis of Mendelian Randomization and Bayesian Colocalization Highlights Four Genes With Putative BMI‐Mediated Causal Pathways to Diabetes.” Scientific Reports 10, no. 1: 7476. 32366963 10.1038/s41598-020-64493-4 PMC7198550

[gepi70037-bib-0058] Mezlini, A. M. , and A. Goldenberg . 2017 October 12. “Incorporating Networks in a Probabilistic Graphical Model to Find Drivers for Complex Human Diseases.” PLOS Computational Biology 13, no. 10: e100558. https://discovery.researcher.life/article/incorporating-networks-in-a-probabilistic-graphical-model-to-find-drivers-for-complex-human-diseases/0e01b96858e23b9eafb4eb64650753d4. 10.1371/journal.pcbi.1005580 PMC5638204 29023450

[gepi70037-bib-0059] Mohammad, G. I. , and T. Michoel . 2024 January 5. “Predicting the Genetic Component of Gene Expression Using Gene Regulatory Networks.” Bioinformatics Advances 4, no. 1: vbae180. 39717201 10.1093/bioadv/vbae180 PMC11665636

[gepi70037-bib-0060] Morrison, J. , N. Knoblauch , J. H. Marcus , M. Stephens , and X. He . 2020 July. “Mendelian Randomization Accounting for Correlated and Uncorrelated Pleiotropic Effects Using Genome‐Wide Summary Statistics.” Nature Genetics 52, no. 7: 740–747. 32451458 10.1038/s41588-020-0631-4 PMC7343608

[gepi70037-bib-0061] Niles, H. E. 1922 May. “Correlation, Causation and Wright's Theory of “Path Coefficients.” Genetics 7, no. 3: 258–273. 17245982 10.1093/genetics/7.3.258 PMC1200533

[gepi70037-bib-0062] Okamoto, J. , X. Yin , B. Ryan , et al. 2025 February 3. “Multi‐Intact: Integrative Analysis of the Genome, Transcriptome, and Proteome Identifies Causal Mechanisms of Complex Traits.” Genome Biology 26, no. 1: 19. 39901160 10.1186/s13059-025-03480-2 PMC11789355

[gepi70037-bib-0063] Park, S. B. , C. K. Chung , E. Gonzalez , and C. Yoo . 2018 November 30. “Causal Inference Network of Genes Related With Bone Metastasis of Breast Cancer and Osteoblasts Using Causal Bayesian Networks.” Journal of Bone Metabolism 25, no. 4: 251–266. 30574470 10.11005/jbm.2018.25.4.251 PMC6288606

[gepi70037-bib-0064] Pividori, M. , S. Lu , B. Li , et al. 2023 September 9. “Projecting Genetic Associations Through Gene Expression Patterns Highlights Disease Etiology and Drug Mechanisms.” Nature Communications 14, no. 1: 5562. 10.1038/s41467-023-41057-4 PMC10492839 37689782

[gepi70037-bib-0065] Porcu, E. , S. Rüeger , K. Lepik , F. A. Santoni , A. Reymond , and Z. Kutalik . 2019a July 24. “Mendelian Randomization Integrating Gwas and eQTL Data Reveals Genetic Determinants of Complex and Clinical Traits.” Nature Communications 10, no. 1: 3300. 10.1038/s41467-019-10936-0 PMC6656778 31341166

[gepi70037-bib-0066] Porcu, E. , S. Rüeger , K. Lepik , F. A. Santoni , A. Reymond , and Z. Kutalik . 2019b July 24. “Mendelian Randomization Integrating GWAS and eQTL Data Reveals Genetic Determinants of Complex and Clinical Traits.” Nature Communications 10, no. 1: 3300. 10.1038/s41467-019-10936-0 PMC6656778 31341166

[gepi70037-bib-0067] Powder, K. E. 2020. “Quantitative Trait Loci (QTL) Mapping.” In eQTL Analysis: Methods and Protocols [Internet], edited by X. M. Shi , 211–229. Springer US. 10.1007/978-1-0716-0026-9_15. 31849018

[gepi70037-bib-0068] Puga, J. L. , M. Krzywinski , and N. Altman . 2015 September 1. “Bayesian Networks.” Nature Methods 12, no. 9: 799–800. 26554085 10.1038/nmeth.3550

[gepi70037-bib-0069] Rancoita, P. M. V. , M. Zaffalon , E. Zucca , F. Bertoni , and C. P. de Campos . 2016 January 1. “Bayesian Network Data Imputation With Application to Survival Tree Analysis.” Computational statistics & data analysis 93: 373–387.

[gepi70037-bib-0070] Rasooly, D. , G. M. Peloso , A. C. Pereira , et al. 2023 July 10. “Genome‐Wide Association Analysis and Mendelian Randomization Proteomics Identify Drug Targets for Heart Failure.” Nature Communications 14, no. 1: 3826. 10.1038/s41467-023-39253-3 PMC10333277 37429843

[gepi70037-bib-0071] Rodríguez‐López, V. , and L. E. Sucar . 2022 April 1. “Knowledge Transfer for Causal Discovery. Int J Approx.” Reason 143: 1–25.

[gepi70037-bib-0072] Sanderson, E. , M. M. Glymour , M. V. Holmes , et al. 2022 February 10. “Mendelian Randomization.” Nature Reviews Methods Primers 2, no. 1: 6. 10.1038/s43586-021-00092-5 PMC7614635 37325194

[gepi70037-bib-0073] Shadrina, A. S. , T. I. Shashkova , A. A. Torgasheva , et al. 2020 June 26. “Prioritization of Causal Genes for Coronary Artery Disease Based on Cumulative Evidence From Experimental and in Silico Studies.” Scientific Reports 10, no. 1: 10486. 32591598 10.1038/s41598-020-67001-w PMC7320185

[gepi70037-bib-0074] Sherif, F. F. , N. Zayed , and M. Fakhr . 2015. “Discovering Alzheimer Genetic Biomarkers Using Bayesian Networks.” Advances in Bioinformatics 2015, no. 1: 639367. 26366461 10.1155/2015/639367 PMC4561111

[gepi70037-bib-0075] Shojaee, A. , and S. C. Huang . 2023 November 1. “Robust Discovery of Gene Regulatory Networks From Single‐Cell Gene Expression Data by Causal Inference Using Composition of Transactions.” Briefings in Bioinformatics 24, no. 6: bbad370. 37897702 10.1093/bib/bbad370 PMC10612495

[gepi70037-bib-0076] Small, D. S. , Z. Tan , R. R. Ramsahai , S. A. Lorch , and M. A. Brookhart . 2017 November. “Instrumental Variable Estimation With a Stochastic Monotonicity Assumption.” Statistical Science 32, no. 4: 561–579.

[gepi70037-bib-0077] Song, M. , J. Greenbaum , J. Luttrell , et al. 2020 October 15. “A Review of Integrative Imputation for Multi‐Omics Datasets.” Frontiers in Genetics 11: 570255. https://www.frontiersin.org/journals/genetics/articles/10.3389/fgene.2020.570255/full. 33193667 10.3389/fgene.2020.570255 PMC7594632

[gepi70037-bib-0078] Subirana‐Granés, M. , J. Hoffman , H. Zhang , et al. 2025 August 11. “Genetic Studies Through the Lens of Gene Networks.” Annual Review of Biomedical Data Science 8(Volume 8, no. 2025: 125–147. 10.1146/annurev-biodatasci-103123-095355 PMC12310179 39977605

[gepi70037-bib-0079] Sun, J. , J. Zhou , Y. Gong , et al. 2024 October 1. “Bayesian Network‐Based Mendelian Randomization for Variant Prioritization and Phenotypic Causal Inference.” Human Genetics 143, no. 9: 1081–1094. 38381161 10.1007/s00439-024-02640-x

[gepi70037-bib-0080] Uffelmann, E. , Q. Q. Huang , N. S. Munung , et al. 2021 August 26. “Genome‐Wide Association Studies.” Nature Reviews Methods Primers 1, no. 1: 59.

[gepi70037-bib-0081] Verbanck, M. , C. Y. Chen , B. Neale , and R. Do . 2018 May. “Detection of Widespread Horizontal Pleiotropy in Causal Relationships Inferred From Mendelian Randomization Between Complex Traits and Diseases.” Nature Genetics 50, no. 5: 693–698. 29686387 10.1038/s41588-018-0099-7 PMC6083837

[gepi70037-bib-0082] Wainberg, M. , N. Sinnott‐Armstrong , N. Mancuso , et al. 2019 April. “Opportunities and Challenges for Transcriptome‐Wide Association Studies.” Nature Genetics 51, no. 4: 592–599. 30926968 10.1038/s41588-019-0385-z PMC6777347

[gepi70037-bib-0083] Wallace, C. 2021 September 29. “A More Accurate Method for Colocalisation Analysis Allowing for Multiple Causal Variants.” PLoS Genetics 17, no. 9: e1009440. 34587156 10.1371/journal.pgen.1009440 PMC8504726

[gepi70037-bib-0084] Wang, G. , A. Sarkar , P. Carbonetto , and M. Stephens . 2020 December. “A Simple New Approach to Variable Selection in Regression, With Application to Genetic Fine Mapping.” Journal of the Royal Statistical Society Series B: Statistical Methodology 82, no. 5: 1273–1300. 37220626 10.1111/rssb.12388 PMC10201948

[gepi70037-bib-0085] Wang, J. , Q. Zhao , J. Bowden , et al. 2021 June 22. “Causal Inference for Heritable Phenotypic Risk Factors Using Heterogeneous Genetic Instruments.” PLoS Genetics 17, no. 6: e1009575. 34157017 10.1371/journal.pgen.1009575 PMC8301661

[gepi70037-bib-0086] Wu, J. , Q. Fan , Q. He , et al. 2023 December 8. “Potential Drug Targets for Myocardial Infarction Identified Through Mendelian Randomization Analysis and Genetic Colocalization.” Medicine 102, no. 49: e36284. 38065874 10.1097/MD.0000000000036284 PMC10713171

[gepi70037-bib-0087] Wu, Y. , K. A. Broadaway , C. K. Raulerson , et al. 2019 December 15. “Colocalization of Gwas and eQTL Signals at Loci With Multiple Signals Identifies Additional Candidate Genes for Body Fat Distribution.” Human Molecular Genetics 28, no. 24: 4161–4172. 31691812 10.1093/hmg/ddz263 PMC7202621

[gepi70037-bib-0088] Xue, H. , X. Shen , and W. Pan . 2021 July 1. “Constrained Maximum Likelihood‐Based Mendelian Randomization Robust to Both Correlated and Uncorrelated Pleiotropic Effects.” American Journal of Human Genetics 108, no. 7: 1251–1269. 34214446 10.1016/j.ajhg.2021.05.014 PMC8322939

[gepi70037-bib-0089] Yang, F. , L. S. Chen , S. Oveisgharan , D. Darbar , and D. A. Bennett . 2024a September. “Integrating Mendelian Randomization With Causal Mediation Analyses FOR Characterizing Direct and Indirect Exposure‐to‐Outcome Effects.” Annals of Applied Statistics 18, no. 3: 2656–2677. 39990115 10.1214/24-aoas1901 PMC11845245

[gepi70037-bib-0090] Yang, S. , J. Guo , Z. Kong , et al. 2024b January 2. “Causal Effects of Gut Microbiota on Sepsis and Sepsis‐Related Death: Insights From Genome‐Wide Mendelian Randomization, Single‐Cell RNA, Bulk RNA Sequencing, and Network Pharmacology.” Journal of Translational Medicine 22: 10. 38167131 10.1186/s12967-023-04835-8 PMC10763396

[gepi70037-bib-0091] Yazdani, A. , A. Yazdani , R. Mendez‐Giraldez , A. Samiei , M. R. Kosorok , and D. J. Schaid . 2022 September 15. “From Classical Mendelian Randomization to Causal Networks for Systematic Integration of Multi‐Omics.” Frontiers in Genetics 13: 990486. https://www.frontiersin.org/journals/genetics/articles/10.3389/fgene.2022.990486/full. 36186433 10.3389/fgene.2022.990486 PMC9520987

[gepi70037-bib-0092] Yin, L. , Y. Feng , Y. Shi , et al. 2024 October. “Estimation of Causal Effects of Genes on Complex Traits Using a Bayesian‐Network‐Based Framework Applied to GWAS Data.” Nature Machine Intelligence 6, no. 10: 1231–1244.

[gepi70037-bib-0093] Yuan, Z. , H. Zhu , P. Zeng , et al. 2020 July 31. “Testing and Controlling for Horizontal Pleiotropy With Probabilistic Mendelian Randomization in Transcriptome‐Wide Association Studies.” Nature Communications 11, no. 1: 3861. 10.1038/s41467-020-17668-6 PMC7395774 32737316

[gepi70037-bib-0094] Zeng, L. , H. A. Talukdar , S. Koplev , et al. 2019 June 18. “Contribution of Gene Regulatory Networks to Heritability of Coronary Artery Disease.” Journal of the American College of Cardiology 73, no. 23: 2946–2957. 31196451 10.1016/j.jacc.2019.03.520 PMC6590059

[gepi70037-bib-0095] Zhang, T. , A. Klein , J. Sang , J. Choi , and K. M. Brown . 2022 June 1. “ezQTL: A Web Platform for Interactive Visualization and Colocalization of QTLs and GWAS Loci.” Genomics, Proteomics & Bioinformatics 20, no. 3: 541–548. 10.1016/j.gpb.2022.05.004 PMC9801033 35643189

[gepi70037-bib-0096] Zhang, W. , T. Lu , R. Sladek , Y. Li , H. Najafabadi , and J. Dupuis . 2024 May 1. “Sharepro: An Accurate and Efficient Genetic Colocalization Method Accounting for Multiple Causal Signals.” Bioinformatics 40, no. 5: btae295. 38688586 10.1093/bioinformatics/btae295 PMC11105950

[gepi70037-bib-0097] Zhao, J. , J. Ming , X. Hu , G. Chen , J. Liu , and C. Yang . 2020 March 1. “Bayesian Weighted Mendelian Randomization for Causal Inference Based on Summary Statistics.” Bioinformatics 36, no. 5: 1501–1508. 31593215 10.1093/bioinformatics/btz749

[gepi70037-bib-0098] Zhao, S. , W. Crouse , S. Qian , K. Luo , M. Stephens , and X. He . 2024 February. “Adjusting for Genetic Confounders in Transcriptome‐Wide Association Studies Improves Discovery of Risk Genes of Complex Traits.” Nature Genetics 56, no. 2: 336–347. 38279041 10.1038/s41588-023-01648-9 PMC10864181

[gepi70037-bib-0099] Zheng, J. , V. Haberland , D. Baird , et al. 2020 October. “Phenome‐Wide Mendelian Randomization Mapping the Influence of the Plasma Proteome on Complex Diseases.” Nature Genetics 52, no. 10: 1122–1131. 32895551 10.1038/s41588-020-0682-6 PMC7610464

[gepi70037-bib-0100] Zhu, A. , N. Matoba , E. P. Wilson , et al. 2021c April 19. “Mrlocus: Identifying Causal Genes Mediating a Trait Through Bayesian Estimation of Allelic Heterogeneity.” PLoS Genetics 17, no. 4: e1009455. 33872308 10.1371/journal.pgen.1009455 PMC8084342

[gepi70037-bib-0101] Zhu, L. , Z. Fang , Y. Jin , et al. 2021a June 27. “Circulating ERBB3 Levels Are Inversely Associated With the Risk of Overweight‐Related Hypertension: A Cross‐Sectional Study.” BMC Endocrine Disorders 21, no. 1: 130. 34176482 10.1186/s12902-021-00793-8 PMC8237455

[gepi70037-bib-0102] Zhu, X. , Z. Duren , and W. H. Wong . 2021b May 14. “Modeling Regulatory Network Topology Improves Genome‐Wide Analyses of Complex Human Traits.” Nature Communications 12, no. 1: 2851. 10.1038/s41467-021-22588-0 PMC8121952 33990562

[gepi70037-bib-0103] Zhu, Z. , F. Zhang , H. Hu , et al. 2016 May. “Integration of Summary Data From Gwas and eQTL Studies Predicts Complex Trait Gene Targets.” Nature Genetics 48, no. 5: 481–487. 27019110 10.1038/ng.3538

[gepi70037-bib-0104] Zhu, Z. , Z. Zheng , F. Zhang , et al. 2018 January 15. “Causal Associations Between Risk Factors and Common Diseases Inferred From Gwas Summary Data.” Nature Communications 9, no. 1: 224. 10.1038/s41467-017-02317-2 PMC5768719 29335400

[gepi70037-bib-0105] Zou, Y. , P. Carbonetto , G. Wang , and M. Stephens . 2022 July 19. “Fine‐Mapping From Summary Data With the “Sum of Single Effects” Model.” PLoS Genetics 18, no. 7: e1010299. 35853082 10.1371/journal.pgen.1010299 PMC9337707

[gepi70037-bib-0106] Zuber, V. , N. F. Grinberg , D. Gill , et al. 2022 May 5. “Combining Evidence From Mendelian Randomization and Colocalization: Review and Comparison of Approaches.” American Journal of Human Genetics 109, no. 5: 767–782. 35452592 10.1016/j.ajhg.2022.04.001 PMC7612737

